# HOW DO SOCIAL NETWORKS CHANGE IN THE LEAD-UP TO DEMENTIA DEATH

**DOI:** 10.1093/geroni/igae098.4339

**Published:** 2024-12-31

**Authors:** Zachary Baker, Andrew Alberth, M Aaron Guest, Allie Peckham, Joahana Segundo, Joseph Saenz

**Affiliations:** Arizona State University, Phoenix, Arizona, United States; University of Massachusetts Boston, Boston, Massachusetts, United States; Arizona State University, Phoenix, Arizona, United States; Arizona State University, Phoenix, Arizona, United States; Arizona State University, Phoenix, Arizona, United States; Arizona State University, Phoenix, Arizona, United States

## Abstract

Larger social networks are associated with a lower risk of dementia, but little is known about how social networks shift when someone has dementia as they approach death. We investigate how social networks among individuals with dementia change as they approach death. We hope this research will inform support systems and care practices that benefit individuals with dementia and their care partners. Participants: 2,295 deceased people with dementia from the Health and Retirement Study (2004-2018). Multilevel models estimated associations between dementia, race/ethnicity, time, and close family and friend network size while controlling for several sociodemographic variables. Social networks shrank linearly as death approached. A decrease in close friends primarily drove shrinkage. Some more unusual patterns emerged when race/ethnicity were crossed with time. Specifically, Hispanic/Latino persons with dementia showed the opposite pattern: as they approached death, the number of close extended family increased dramatically, an increase of one person every two and a half years. Dementia risk, social networks, and patterns of social network shrinking are unequal across people of different races and ethnicities. Contrasting patterns of increasing numbers of extended may be driven by familismo. Perhaps as the needs of the people with dementia increased, family members “rallied around” to provide care. Given the importance of social networks, especially during challenging times, and their tendency to shrink closer to death, if non-Hispanic/Latino individuals could adopt the effective strategies used by Hispanic/Latino older adults to maintain and grow their social networks, they might experience similar benefits of robust social networks.

